# Chronic Atrophic Orchitis in a Bull1Presented at the Fourteenth Annual Meeting of the Iowa State Veterinary Medical Association, Des Moines, Iowa, February 11 and 12, 1902.

**Published:** 1902-04

**Authors:** H. C. Simpson

**Affiliations:** Denison, Iowa


### CHRONIC ATROPHIC ORCHITIS IN A BULL.[1]

By H. C. Simpson, D.V.S.,
DENISON, IOWA.

In the spring of 1901 I was called to see a valuable bull, Tenth Laird of Estell, property of Mr. McHenry. Accompanied by Dr. Gibson I examined the animal, and found the left side of the scrotum enlarged. It was about eight inches in diameter at the base, and on warm days it was said to hang nearly to the ground. It was hard, tense, and not tender to pressure. The right side of the scrotum and the right testicle appeared normal, except that it also was hanging somewhat lower, the other side pulling it down.

The cause assigned for this condition was that the bull was supposed to have been kicked by a negro attendant about one year previously. The animal had been shown the previous summer with this enlarged testicle at a number of State fairs, and had won continuously; but the condition was fast becoming worse, and it was evident that something had to be done. On account of the value of the Tenth Laird—Mr. McHenry having paid $1200 for him—he hesitated about advising castration, as neither of us had heard of a case where the animal's usefulness as a sire was preserved after removal of one testicle, so at that time we advised iodide of potassium, one drachm twice daily, until iodism was produced, the administration then to be discontinued and resumed again shortly. No corn was to be fed while under treatment. An iodine ointment was also to be applied twice daily externally and well rubbed in. The ointment was made by rubbing up the iodine crystals in sulphuric ether and then mixing with lard. This form of medication was given a thorough trial, but with no good

[1] Presented at the Fourteenth Annual Meeting of the Iowa State Veterinary Medical Association, Des Moines, Iowa, February 11 and 12, 1902.

results that we could see, so it was decided to operate. The animal was prepared by dieting a few days. Owing to his size—2100 pounds—we were at a loss to know how to cast him, but finally decided to use an ordinary casting harness and side lines. In addition to this we looped a rope around his body, all of which came in handy, as the animal broke the harness. There was plenty of help, but he gave all of us a tussle before he went down. The scrotum and surrounding parts were thoroughly disinfected with a chloronaptholeum solution. The instruments were immersed in a bucket of the same solution. A puncture was made through the skin near the body, and a trocar and canula inserted full length toward the external inguinal ring, but nothing was found. The skin was normal, but the subjacent tissues were very much thickened, there being one and one-half inches of newly-formed tissue to go through before reaching the testicle. The connective tissue around the testicle was broken down with the hands. One end of the écraseur chain was put around the cord as high up as possible, and after rendering fairly tight, the cord, which was over one inch in diameter, was ligated; then the chain was loosened and slipped down toward the testicle a short distance, and again tightened until the cord was cut in two. Afterward all loose tissue was removed, the wound was washed out thoroughly, packed with cotton saturated in chloro-naptholeum solution, and the animal allowed to rise. After-treatment consisted in daily flushing out with chloronaptholeum solution. The wound healed well. The other side of the scrotum has contracted to normal shape, and a person seeing the bull now would never know that anything had been wrong with him. In regard to his breeding it may be said that he had sired four good, strong, healthy calves before being operated upon. Last autumn after the operation he served a number of cows, and has apparently impregnated them all, as none has come into heat since being served.

The testicle was packed in a box with ice and shipped to Dr. Repp, at the Iowa State College, who has examined it and can give the results of his examination.

### Discussion.

Dr. Neiman said that he has under his care a bull suffering in a way similar to Dr. Simpson's case, but that he has not been able to obtain the owner's consent to castration.

Dr. Repp described the microscopical appearance of the testicle and the capsule of connective tissue surrounding it. He stated that the disease was neither actinomycosis nor tuberculosis.

Dr. Gibson described the operation, saying that it was much more difficult than castration when the testicles are normal.

Dr. Heck said that a steer under his care suffered from a necrotic process involving the sheath and skin of the abdomen, which he thought was induced by foulness of the sheath. As a result the sheath and an area of skin about fifteen inches in diameter became separated and was cast off. This mass weighed over thirty pounds. The wound healed, and the steer was sent to market in good condition a month later. The scar was hardly noticeable.

Dr. C. E. Stewart had a similar case.

Dr. D. H. Miller related an experience with abscess of the sheath of a bull which extended to the testicles and set up an acute orchitis. Relief was had by opening the abscess.

